# Supplementary material for: Clerodendranthus spicatus (Thunb.) Water Extracts Reduce Lipid Accumulation and Oxidative Stress in the Caenorhabditis elegans
Source: Int J Mol Sci. 2024 Sep 6;25(17):9655. doi: 10.3390/ijms25179655 (PMC11394974; doi:10.3390/ijms25179655)
Supplement: Supplementary file 1 [file ijms-25-09655-s001.zip › ijms-3188640-supplementary.pdf]

Supplementary data

Table S1 Effects of CSw on the lifespan of *C. elegans*.

Strain(solvent)	Maximum lifespan(d)	Mean lifespan (d)(±SEM)	p value versus control
N2 (Control)	23	17.00±0.89	
N2 (20 μg/mL)	29	21.46±0.71	＜0.01
N2 (50 μg /mL)	28	20.63±0.77	＜0.05
N2 (100 μg / mL)	26	19.40±0.73	
N2 (500 μg / mL)	24	18.46±0.62	
N2 (1000 μg / mL)	25	18.33±0. 88	

Table S2 Preliminary identification of derivatives extracted from CSw.

No.	ID	RT/min	Precursorion	Meas.m/z	m/z	Error/ppm	Formula	Identification
Phenolic acids								
1	M183T116	1.93	$[M+H]^+$	183.0613	182.0579	21.17	$C_9H_{10}O_4$	Homovanillic acid
2	M181T572	9.53	$[M+H]^+$	181.0751	180.0786	16.54	$C_{10}H_{12}O_3$	Coniferyl alcohol
3	M207T587	9.78	$[M+H]^+$	207.0656	224.0685	21.89	$C_{11}H_{12}O_5$	Sinapic acid
4	M179T202	3.36	$[M+H]^-$	179.0359	180.0423	4.89	$C_9H_8O_4$	Caffeate
5	M181T204	3.39	$[M+H]^-$	181.0506	182.0579	0.00	$C_9H_{10}O_4$	3,4-Dihydroxyhydrocinnamic acid
6	M193T345	5.74	$[M+H]^-$	193.051	194.0579	1.94	$C_{10}H_{10}O_4$	trans-Ferulic acid
7	M193T345	5.74	$[M+H]^-$	193.051	194.0579	1.94	$C_{10}H_{10}O_4$	Ferulic acid
8	M163T378	6.30	$[M+H]^-$	163.0406	164.0473	3.53	$C_9H_8O_3$	4-Hydroxycinnamic acid
9	M197T422	7.02	$[M+H]^-$	197.0433	198.0528	6.09	$C_9H_{10}O_5$	Syringic acid
10	M167T528	8.80	$[M+H]^-$	167.0359	168.0423	5.24	$C_8H_8O_4$	Vanillic acid

No.	ID	RT/min	Precursorion	Meas.m/z	m/z	Error/ppm	Formula	Identification
11	M359T569	9.47	$[M+H]^-$	359.0785	360.0485	3.55	$C_{18}H_{16}O_8$	Rosmarinic acid
12	M169T826	13.77	$[M+H]^-$	169.0159	170.0215	9.91	$C_7H_6O_5$	Gallic acid
Flavonoids								
13	M449T480	7.99	$[M+H]^+$	449.1121	449.1084	7.42	$C_{21}H_{21}O_{11}$	Cyanidin 3-glucoside
14	M303T508	8.46	$[M+H]^+$	303.0502	302.0430	0.25	$C_{15}H_{10}O_7$	Quercetin
15	M287T632	11.36	$[M+H]^+$	287.0548	286.0480	10.52	$C_{15}H_{10}O_6$	Fisetin
16	M271T682	11.36	$[M+H]^+$	271.0603	270.0528	0.44	$C_{15}H_{10}O_5$	Apigenin
17	M301T376	12.27	$[M+H]^+$	301.0707	300.0634	0.07	$C_{16}H_{12}O_6$	Kaempferide
18	M373T818	13.62	$[M+H]^+$	373.1284	372.1209	0.53	$C_{20}H_{20}O_7$	Tangeritin
19	M373T978	16.30	$[M+H]^+$	373.1360	372.1209	20.90	$C_{20}H_{20}O_7$	Sinensetin
20	M299T461	7.68	$[M+H]^-$	299.0567	300.0634	1.92	$C_{16}H_{12}O_6$	Diosmetin
21	M271T466	11.26	$[M+H]^-$	271.0617	272.0685	1.75	$C_{15}H_{12}O_5$	Naringenin

No.	ID	RT/min	Precursorion	Meas.m/z	m/z	Error/ppm	Formula	Identification
22	M270T677	11.28	$[M+H]^-$	270.0493	270.0528	2.50	$C_{15}H_{10}O_5$	Genistein
23	M315T702	11.70	$[M+H]^-$	315.0540	316.0583	9.43	$C_{16}H_{12}O_7$	Isorhamnetin
Terpenoids								
24	M151T73	1.21	$[M+H]^+$	151.0354	150.0528	2.97	$C_5H_{10}O_5$	D-Ribose
25	M162T97	1.61	$[M+H]^+$	162.0762	161.1052	29.45	$C_7H_{15}NO_3$	L-Carnitine
26	M118T126	2.10	$[M+H]^+$	118.0862	117.0790	0.64	$C_5H_{11}NO_2$	Betaine
27	M175T143	2.38	$[M+H]^+$	175.1080	174.1004	1.71	$C_7H_{14}N_2O_3$	L-Theanine
28	M114T284	4.73	$[M+H]^+$	113.9635	113.0589	4.36	$C_4H_7N_3O$	Creatinine
29	M180T489	7.97	$[M+H]^+$	180.0658	197.0688	24.62	$C_9H_{11}NO_4$	L-Dopa
30	M183T990	16.49	$[M+H]^+$	182.9855	182.0790	3.30	$C_6H_{14}O_6$	Sorbitol
31	M341T116	1.94	$[M+H]^-$	341.1087	342.1162	0.21	$C_{12}H_{22}O_{11}$	Trehalose
32	M271T169	2.81	$[M+H]^-$	271.0831	272.0896	2.85	$C_{12}H_{16}O_7$	Arbutin

No.	ID	RT/min	Precursorion	Meas.m/z	m/z	Error/ppm	Formula	Identification
33	M124T290	4.83	$[M+H]^-$	124.0409	125.0147	15.63	$C_2H_7NO_3S$	Taurine
34	M473T298	4.96	$[M+H]^-$	473.0735	474.0498	2.00	$C_{22}H_{18}O_{12}$	Chicoric acid
35	M503T546	9.09	$[M+H]^-$	503.1768	504.1690	29.81	$C_{18}H_{32}O_{16}$	Raffinose
Prenol lipids								
36	M235T613	10.21	$[M+H]^+$	235.1696	134.1620	1.37	$C_{15}H_{22}O_2$	Curcumenol
37	M203T782	13.03	$[M+H]^+$	203.1796	220.1827	21.33	$C_{15}H_{24}O$	Caryophyllene alpha-oxide
38	M439T818	13.62	$[M+H]^+$	439.3555	456.36.3	5.99	$C_{30}H_{48}O_3$	Oleanolic acid
39	M551T861	14.35	$[M+H]^+$	551.4326	568.4280	21.82	$C_{40}H_{56}O_2$	Lutein
Fatty Acyls								
40	M200T990	16.49	$[M+H]^+$	199.9759	200.1776	3.85	$C_{12}H_{24}O_2$	Dodecanoic acid
41	M255T784	13.07	$[M+H]^-$	255.0726	256.2402	0.77	$C_{16}H_{32}O_2$	Palmitic acid
	Coumarins and derivatives							

No.	ID	RT/min	Precursorion	Meas.m/z	m/z	Error/ppm	Formula	Identification
42	M161T698	11.63	$[M+H]^{-}$	161.0252	162.0317	4.81	$C_9H_6O_3$	Umbelliferone
43	M177T726	12.09	$[M+H]^{-}$	177.0211	178.0266	9.98	$C_9H_6O_4$	Aesculetin

8 Table S3 The list of primer.

Gene	Primer（5’ to 3’）
daf-16	CCAGACGGAAGGCTTAAACT
	ATTCGCATGAAACGAGAATG
skn-1	AGTGTCGGCGTTCCAGATTTC
	GTCGACGAATTGCGAATCA
hsf-1	GAATGCGACTAGGCAAATGGC
	GGTGGATGAGGTGGAAGTCG
nhr-49	AGGCTCGTGTCAATCAAGAGATGTG
	ATGCCGATGCTCCAGAATCACTTC
fat-7	CAACAGCGCTGCTCACTATT
	CACCAACGGCTACAACTGTG
acs-2	GCAGCCTCGCTCTACACTCT
	GACTCCTGCAAATGCACATGC
ech-1	CGTCGGAGCTGGATTCATGG
	TCGTTCAACGCCTGCCTGGT
sod-3	CTCCAAGCACACTCTCCCAG
	TCCCTTTCGAAACAGCCTCG
ctl-1	GACGTATCCAAAACCCCAAGTG
	TTGGCATGAACGACACGCTC
ctl-2	TTCCGATCGAGGACTCCCAG
	CTTCACTCCTTGAGTTGGCTTG
ctl-3	CCCACATGGTCAATCTAACGGT
	GGAGCTCCATTGGATGTGGT
hsp-16.2	CTGCAGAATCTCTCCATCTGAGTC
	AGATTCGAAGCAACTGCACC
gcs-1	GTCGATGAAGCCAGATGGTTGT
	CGATCGTCGACACTTGCACTAA
gst-4	ATGCTCGTGCTCTTGCTGAG

Gene	Primer（5’ to 3’）
	GACTGACCGAATTGTTCTCCAT
gpd-1	GGAAACATCTGGTGTAGGGA
	CACTGGCGAAGGTTATCAA
daf-2	GTACAGCCGTGTGCCTCAATAGTC
	ATTGTCAGCGAACCTTCCACCAC
age-1	GCTGCTCCGTGCAGAGATTG
	CACGGAGGTAAGCTTCCATC
daf-15	GTCGAAGCAGGAATGCCCGA
	CGTGGCATTTCGACAGTCGC
ogt-1	ACCTCCGCCGATTCCTGTGATC
	CAACTTGCCGTTGAGCATTCATAAGG
oga-1	ATCATTCAGTCGGCGGTTGC
	TCCACAGGATCTTCCGTTTC
sbp-1	CACCACCTCATCACCACCATCAC
	TTCGTCTCTGGAGCATCTTCAATCG
mdt-15	ACCAGCAATGGGTCAGCCAC
	GAGCACCACTACCAGGTGGT
fat-5	CGGCCGCCCTCTTCCGTTAC
	TGGCTGCCATCCGACCCAGT
fat-6	TCAACAGCGCTGCTCACTAT
	TTCGACTGGGGTAATTGAGG

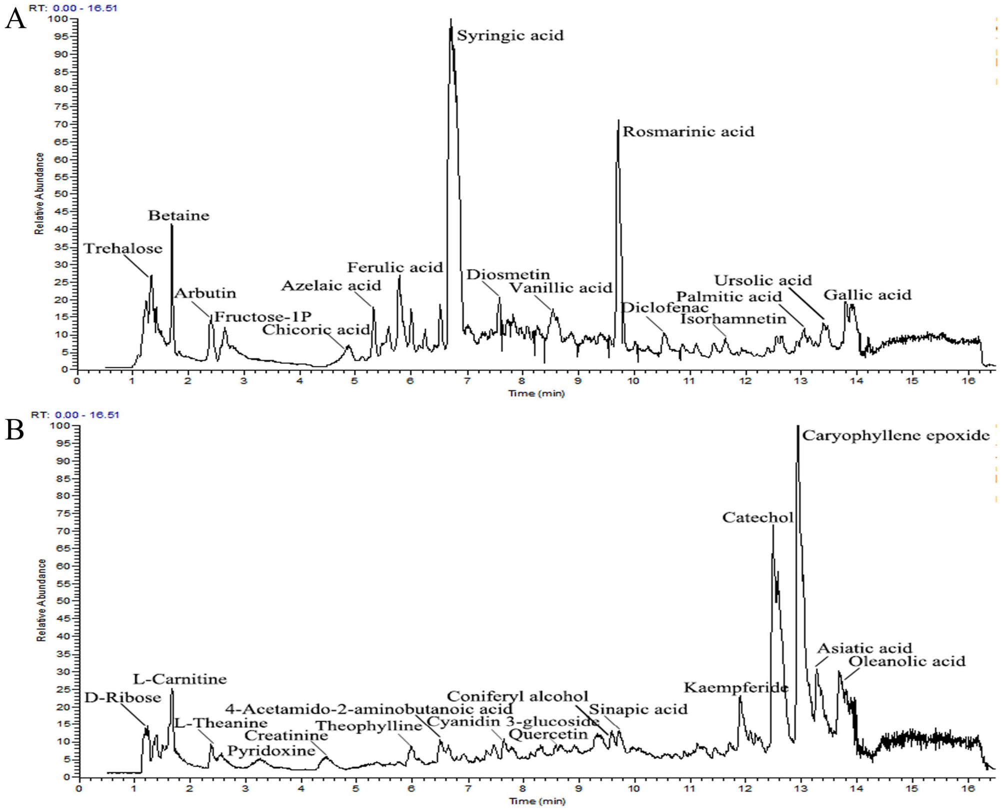

Figure S1. CSw chemical composition analysis. (A) Negative ion mode chromatogram; (B) Positive ion mode chromatogram.
